# Comparative analysis of diagnostic accuracy between transoral and submental contrast-enhanced ultrasound-guided biopsy for tonsillar tumors

**DOI:** 10.3389/fonc.2025.1513756

**Published:** 2025-09-05

**Authors:** Ting Wei, Man Lu, Tingting Li, Ziyue Hu, Lu Wang, Juan Li, Jiulong Dai, Xiaobo Wu, Bo Tan, Fawei He, Yuan Li, Wei Yang

**Affiliations:** Department of Ultrasound Medical Center, Sichuan Clinical Research Center for Cancer, Sichuan Cancer Hospital & Institute, Sichuan Cancer Center, University of Electronic Science and Technology of China, Chengdu, China

**Keywords:** ultrasonography, biopsy, tonsil neoplasms, needle biopsy, transoral approach, submental approach

## Abstract

**Objective:**

To assess and compare the diagnostic accuracy and safety of contrast-enhanced ultrasound (CEUS)-guided transoral and submental core needle biopsy (CNB) techniques in patients with suspected tonsillar masses, and to identify which approach offers superior diagnostic performance with fewer complications.

**Methods:**

Between November 2019 and March 2024, 41 patients with suspected tonsillar masses were enrolled in this comparative study of two biopsy techniques. Each patient underwent either a transoral CNB or a submental CNB. Diagnostic metrics, including accuracy, sensitivity, negative predictive value (NPV), and positive predictive value (PPV) were calculated for each method. Pain levels before and after the procedures were recorded to evaluate patient discomfort, and any complications were documented to evaluate safety. Statistical analyses were conducted to determine whether differences in diagnostic performance, biopsy time, and complication rates between the two techniques were significant.

**Results:**

A total of 41 patients were included in the analysis (transoral CNB, n = 22; submental CNB, n = 19). The transoral approach demonstrated higher diagnostic accuracy (95.45% vs. 89.47%), sensitivity (91.67% vs. 87.5%), and NPV (90.91% vs. 60.00%) compared with the submental approach; PPV was 100% for both methods. Pre- and post-biopsy pain scores were similar in both groups, with no significant differences observed. No significant complications occurred in the transoral group. In the submental group, two patients developed minor acute submandibular adenitis; however, this difference in complication rates (0% vs. 10.5%) was not statistically significant (P = 0.21).

**Conclusion:**

The transoral CEUS-guided CNB approach demonstrated higher diagnostic accuracy and lower post-procedural complication rates than the submental method in the evaluation of tonsillar tumors. These findings support the transoral route as the preferred technique for obtaining tonsillar biopsy specimens and may inform clinical practice changes aimed at improving patient outcomes through more accurate and timely diagnosis.

## Introduction

Tonsillar tumors are a significant clinical concern within the field of head and neck oncology, contributing substantially to both morbidity and mortality associated with oral cancers. Prompt and accurate diagnosis is essential, as diagnostic delays are closely linked to more advanced disease at presentation and poorer survival outcomes (1–5). Conventional diagnostic modalities, including direct visual inspection, CT and MRI imaging (6), and histopathological evaluation through biopsy, each have inherent limitations. Visual examination and palpation may fail to detect small, deeply situated, or submucosal lesions, while imaging alone cannot provide the cellular architecture necessary for definitive diagnosis and treatment planning.

Although histopathological analysis remains the gold standard for diagnosis, traditional incisional biopsy performed under direct visualization is prone to false-negative results due to insufficient or non-representative tissue sampling, particularly in anatomically complex or submucosal regions (7, 8).

To address these challenges, ultrasound-guided core needle biopsy (CNB) has gained increasing clinical adoption as a minimally invasive and reliable alternative for evaluating oropharyngeal lesions (9, 10). Ultrasound technology enables real-time visualization of lesion morphology, needle trajectory, and adjacent anatomical structures, thereby improving the precision of tissue acquisition compared to traditional methods (9).

Nevertheless, conventional B-mode ultrasound has limitations in distinguishing viable tumor tissue from adjacent inflammatory or necrotic areas, which can compromise biopsy accuracy—particularly in tumors with heterogeneous echotexture or poor vascularity (9). Contrast-enhanced ultrasound (CEUS), which employs intravenous microbubble contrast agents, addresses these limitations by improving the visualization of tumor perfusion and vascular architecture. CEUS enhances the delineation between viable and non-viable regions by providing dynamic, real-time assessment of microvascular blood flow within the lesion (9). This is particularly beneficial in guiding biopsy needle placement, as viable tissue is more likely to yield diagnostically adequate samples (9). Furthermore, CEUS can reduce sampling errors and avoid necrotic zones, which are common in larger or advanced tumors (9).

Currently, two primary ultrasound-guided biopsy routes—transoral and submental—are utilized in clinical practice for sampling tonsillar tumors. The existing literature on ultrasound-guided CNB for tonsillar tumors is limited, with most studies based on small patient cohorts. Moreover, no studies to date have compared the diagnostic performance and safety of transoral versus submental approaches under CEUS guidance for tonsillar tumors. Given the anatomical and technical distinctions between these two approaches, establishing the optimal access route is clinically important. This study aims to compare the diagnostic accuracy, procedural safety, and technical feasibility of CEUS-guided transoral versus submental CNB in patients with tonsillar tumors.

## Materials and methods

### Patients

This retrospective comparative study was approved by the institutional review board and ethics committee of Sichuan Cancer Hospital (Grant Number JS - 2018-022-01). All participants provided written informed consent prior to inclusion in the study. Between November 2019 and March 2024, 41 patients were analyzed in this study. Inclusion criteria were patients presenting with palpable or radiologically detected tonsillar masses and confirmed diagnosis by surgical resection pathology. Exclusion criteria included patients with known bleeding disorders, those unable to undergo CEUS due to contrast agent allergy, those with poor general health precluding biopsy procedures, and those without subsequent surgical resection and pathological confirmation.

### Pre-biopsy evaluation

Prior to the biopsy procedure, all patients underwent a comprehensive ultrasound evaluation. B-mode ultrasound was initially performed to assess morphologic features of lesions including echogenicity, size, and margin. Color Doppler imaging was used to evaluate internal vascularity and adjacent vasculature. Subsequently, the contrast harmonic imaging mode was activated. CEUS was conducted to further characterize tumor perfusion and guide the biopsy trajectory. A 2.4-mL bolus of SonoVue^®^ (Bracco, Milan, Italy) was administered intravenously via the antecubital vein, immediately followed by a 5-mL saline flush. CEUS imaging was performed in real time to observe the contrast enhancement pattern during the arterial phase. Areas exhibiting strong enhancement were identified as viable tumor tissue, while non-enhancing zones were presumed necrotic (9). The transducer position and needle guidance line were adjusted to ensure that the biopsy path traversed enhancing, viable regions and avoided non-enhancing necrotic zones.

### Biopsy procedure

CEUS-guided transoral CNB and submental CNB were performed by an experienced radiologist (M.L., with 20 years of experience in musculoskeletal US, CEUS, and intervention).

### Transoral approach

**Figure 1:**
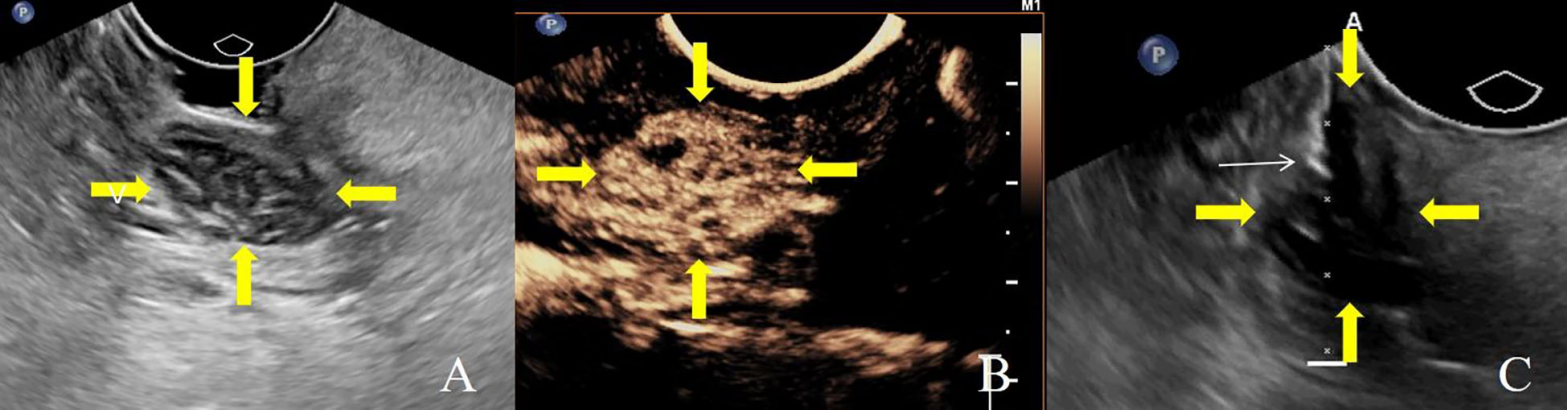
A 52-year-old female patient with left tonsil enlargement, underwent transoral core needle biopsy. **(A)** Transoral ultrasound shows enlarged tonsil volume with decreased echogenicity. **(B)** Contrast-enhanced ultrasound during the arterial phase shows high enhancement. **(C)** Ultrasound-guided transoral core needle biopsy. Histological diagnosis confirmed reactive hyperplasia of the tonsil. Yellow arrows indicate the tumor edge, and white arrows indicate the biopsy needle.

Patients were positioned in the supine decubitus position and received local anesthesia via 10 mL of lidocaine hydrochloride mucilage to ensure comfort throughout the procedure. Ultrasound imaging was performed using a high-frequency 10 MHz endocavitary transducer (Philips EPIQ 7, Bothell, WA) equipped with a sterile probe cover filled with ultrasonic gel to maintain sterility and optimize image quality. Color Doppler and intravenous contrast-enhanced sonography were conducted to identify viable tissue and avoid critical anatomical structures such as blood vessels and nerves. An electronic biopsy line was activated to visualize the needle path in real time. Biopsies were executed using an 18-gauge automatic core biopsy needle with a 15 or 22-mm throw (Magnum and Max-Core, Bard, Tempe, AZ, USA), chosen based on lesion size and location. The needle was advanced under continuous ultrasound guidance to ensure accurate placement within enhancing tissue (**Figure 1**). Three tissue samples were routinely collected, each considered adequate if exceeding 0.5 cm in length, and were immediately fixed in 10% neutral buffered formalin for pathological analysis. Post-procedure, manual compression with gauze was applied at the biopsy site for 3 minutes to control bleeding.

### Submental approach

**Figure 2:**
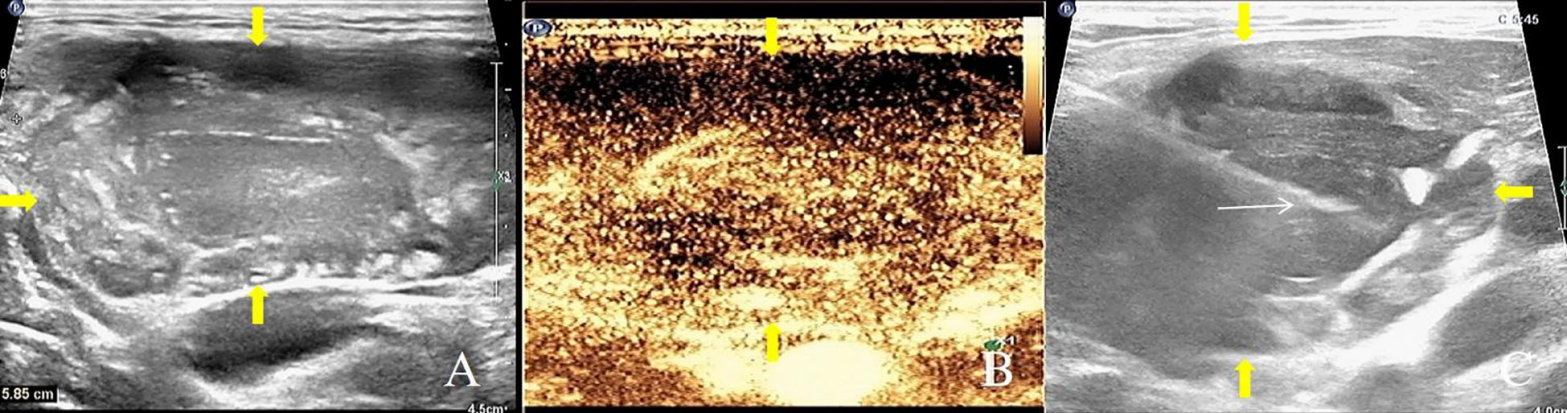
A 53-year-old male patient with left tonsil enlargement, underwent submental core needle biopsy. **(A)** Submental ultrasound indicates a hypoechoic mass in the left tonsil with unclear borders and irregular shape. **(B)** Contrast-enhanced ultrasound during the arterial phase shows rapid and high enhancement. **(C)** Ultrasound-guided submandibular core needle biopsy. Histological diagnosis confirmed tonsillar lymphoma. Yellow arrows indicated the edge of tumor; white arrows indicated the biopsy needle. Yellow arrows indicate the tumor edge, and white arrows indicate the biopsy needle.

The patient was positioned supine with the neck extended as tolerated to adequately expose the submental region. A 5 – 12 MHz linear probe (Philips EPIQ 7, Bothell, WA) was used to optimize visualization of lesions located between the hyoid bone and mandible. B-mode ultrasound, color Doppler, and intravenous contrast-enhanced sonography were performed to evaluate the morphology of the lesion and its spatial relationship to adjacent anatomical structures. The biopsy area was prepared aseptically, and 2 mL of 2% lidocaine was administered along the planned biopsy trajectory for local anesthesia. Biopsy was carried out using the same 18-gauge automatic core biopsy needle as in the transoral approach, with the needle inserted under continuous ultrasound guidance following the planned trajectory beneath the skin. Tissue samples were obtained sequentially (**Figure 2**). Similar to the transoral technique, three samples were routinely collected and immediately preserved for pathological examination. Manual compression was applied post-procedure to control bleeding and ensure hemostasis.

### Post-biopsy evaluation

The core specimens retrieved from each biopsy were sent for histopathological examination. The primary endpoint was the diagnostic accuracy of each approach, measured as the congruence between biopsy-provided diagnoses and the final diagnoses established from surgical specimens or comprehensive clinical evaluation.

Biopsy time was defined as the duration from the insertion of the biopsy needle for the first puncture to the removal of the needle after the final tissue sample was obtained. The biopsy time for each biopsy was recorded in seconds. All procedural complications were recorded. Major complications included any event requiring therapeutic intervention, prolongation of hospitalization (> 24 h), permanent morbidity, or death. Minor complications were defined as self-limited events requiring no treatment or only short-term observation (e.g., mild bleeding, transient nerve impairment, mild infection, or submandibular adenitis). The incidence of complications was documented and compared between the two techniques. Patient tolerability was assessed using a visual analogue scale for pain before and immediately after the procedure.

### Statistical analysis

All data analyses were performed using the statistical software SPSS version 25. Normality of continuous variables was assessed using the Shapiro-Wilk test. Continuous variables were expressed as mean ± standard deviation (SD) and compared using the independent samples t-test. Categorical variables were summarized as counts and percentages and analyzed using the chi-square test or Fisher’s exact test. Diagnostic performance metrics, including diagnostic accuracy, sensitivity, specificity, positive predictive value (PPV), and negative predictive value (NPV) were calculated for each approach. A p-value of less than 0.05 was considered statistically significant.

## Results

This study enrolled 41 patients with clinically or radiologically suspected tonsillar masses. The baseline characteristics of these patients are summarized in **Table 1**. Of these patients, 22 underwent transoral CNB, and 19 underwent submental CNB. The transoral biopsy approach demonstrated higher diagnostic accuracy (95.45%) compared with the submental approach (89.47%). The transoral approach also showed greater sensitivity (91.67% vs. 87.5%) and a higher negative predictive value (90.91% vs. 60.00%). Both approaches achieved a positive predictive value of 100% (**Table 2**).

**Table 1:** Patient and lesion characteristics.

Characteristics	Transoral approach (N = 22)	Submental approach (N = 19)	P value^*^
Age (Mean± standard), years^†^	56.41 ± 12.56	55.95 ± 14.02	0.71
Male/Female	10/12	13/6	0.25
Size of tumor (Mean± standard), mm^†^	21.86 ± 7.91	28.79 ± 9.43	0.01

Unless otherwise noted, data are numbers of patients or lesions.

^†^Data are mean ± standard deviations.

^*^Independent sample T-test was used for comparisons of quantitative variables. Pearson Chi-square test and Fisher’s exact test were applied for comparisons of categorical variables.

**Table 2:** Diagnostic yield compared to final histology.

	Transoral approach			Submental approach		
	Malignant	Benign	Total	Malignant	Benign	Total
Malignant at definitive final diagnosis	11	1	12	14	2	16
Benign at definitive final diagnosis	0	10	10	0	3	3
Total	11	11	22	14	5	19

Unless otherwise noted, data are numbers of patients or lesions.

**Figure 3:**
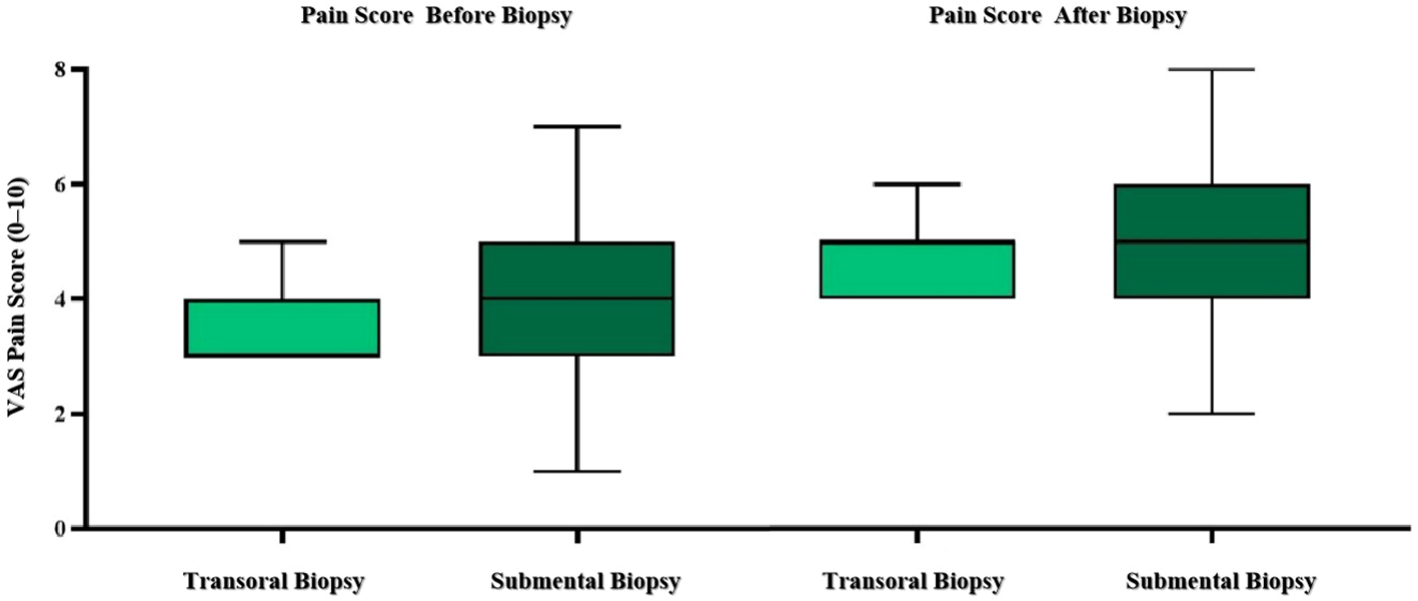
Pain score before and after biopsy in transoral and submental CEUS guided biopsy for tonsillar tumors.

Regarding patient discomfort, pain scores assessed before and after the biopsy procedures showed no significant differences between the transoral and submental approaches. Pain scores were comparable between the transoral and submental approaches before biopsy (3.77 ± 1.19 vs. 4.37 ± 1.80, P = 0.21) and after biopsy (5.05 ± 1.09 vs. 5.26 ± 1.82, P = 0.64) (**Figure 3**). Biopsy time was also comparable (64 ± 5 seconds for transoral vs. 66 ± 4 seconds for submental, P = 0.13). Tumor size in the transoral group averaged 21.86 ± 7.91 mm, whereas in the submental group it averaged 28.79 ± 9.43 mm. Tumor size differed significantly between the transoral and submental groups (P = 0.01).

**Figure 4:**
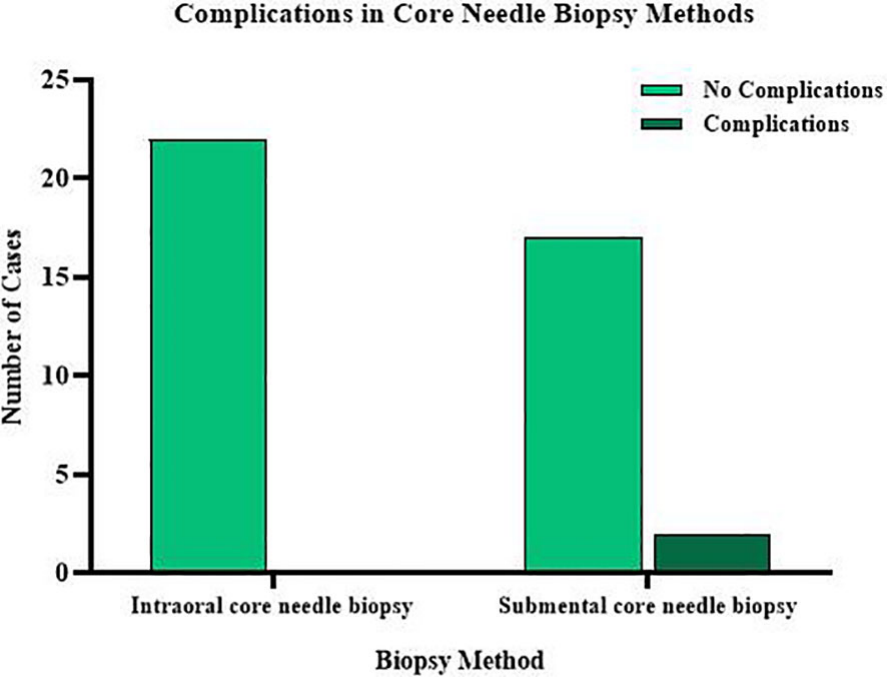
Complications in transoral and submental CEUS guided biopsy for tonsillar tumors.

Safety profiles for both procedures were favorable, with no severe complications noted. The transoral approach did not result in any major complications, while the submental approach experienced two minor events of acute submandibular adenitis (**Figure 4**). The difference in complication rates between the two methods was not statistically significant (0% for transoral; 10.5% for submental, P = 0.21).

## Discussion

This study provides a comparison between the transoral and submental CEUS-guided CNB techniques for diagnosis of tonsillar masses. Our results demonstrate that the transoral approach offers superior diagnostic accuracy (95.45% vs. 89.47%) and sensitivity (91.67% vs. 87.5%) compared to the submental approach. This advantage is likely due to the ability of the transoral technique to directly access the tonsillar tissue under real-time visualization, thereby reducing sampling errors that are more common with the indirect submental route. Additionally, CNB specimens enabled immunohistochemical testing to guide therapeutic decisions.

Both biopsy approaches exhibited favorable safety profiles, with minimal complications reported. However, two minor cases of acute submandibular adenitis occurred in the submental group, possibly related to the trajectory of the biopsy needle in close proximity to the submandibular gland. This finding underscores the necessity of meticulous procedural planning and needle guidance to mitigate glandular irritation or injury during the submental approach.

Pre-procedural pain levels were elevated in some patients, attributable to inflammation, ulceration, local mass effect, and proximity of the tumor to nerve−rich regions. Post-biopsy, only a mild increase in discomfort was observed, with no statistically significant difference between the two groups. These results suggest that CEUS-guided biopsy is generally well tolerated, with post-procedural pain remaining within clinically acceptable limits.

Tumor size differed between groups, with larger lesions more frequently biopsied via the submental route. Smaller lesions posed challenges for submental ultrasound visualization, increasing risks of inaccurate needle placement and potential injury to surrounding structures. In contrast, intraoral ultrasound provided superior image resolution and lesion delineation, facilitating precise targeting and reducing procedural risks. Consequently, the transoral approach may be preferable for small or anatomically challenging lesions to optimize diagnostic yield and patient safety.

The use of CEUS substantially enhanced biopsy efficacy by improving visualization of tumor vascularity and boundaries. CEUS allowed differentiation of viable tumor tissue from necrotic or non-representative areas, mitigating sampling errors inherent to conventional ultrasound guidance. Benign tonsillar lesions typically demonstrated homogeneous enhancement patterns, whereas malignant lesions exhibited heterogeneous enhancement with irregular margins and rapid contrast wash-in and wash-out dynamics. These enhancement patterns reflect the high level of angiogenesis in malignant tumors and offer valuable diagnostic cues to distinguish malignant from benign pathology.

This study has several limitations. Its retrospective, single-center design may limit the generalizability of the findings. Additionally, operator expertise in both CEUS imaging and biopsy technique could influence diagnostic outcomes and introduces potential variability.

In summary, our findings support the clinical utility of transoral CEUS-guided CNB as a highly accurate and safe technique for evaluating tonsillar tumors. The superior visualization afforded by CEUS and the direct access of the transoral approach contribute to improved diagnostic performance. Broader adoption of this technique, where clinically feasible, may enhance diagnostic accuracy and patient experience in the management of tonsillar masses.

## Data Availability

The raw data supporting the conclusions of this article will be made available by the authors, without undue reservation.
